# Host immune response–inspired development of the influenza vaccine

**DOI:** 10.1016/j.anai.2020.04.008

**Published:** 2020-07

**Authors:** Angela Choi, Adolfo García-Sastre, Michael Schotsaert

**Affiliations:** ∗ Graduate School of Biomedical Sciences, Icahn School of Medicine at Mount Sinai, New York, New York; † Global Health and Emerging Pathogens Institute, Icahn School of Medicine at Mount Sinai, New York, New York; ‡ Department of Medicine, Division of Infectious Diseases, Icahn School of Medicine at Mount Sinai, New York, New York; § The Tish Cancer Institute, Icahn School of Medicine at Mount Sinai, New York, New York; ¶ Department of Microbiology, Icahn School of Medicine at Mount Sinai, New York, New York

## Abstract

**Objective:**

To assess the current and future development of influenza vaccines.

**Data Sources:**

PubMed searches were performed cross-referencing the keywords influenza, influenza vaccine, host immune response, correlates of protection, vaccine development, vaccine efficacy. Articles were reviewed for additional citations.

**Study Selections:**

Articles were reviewed and selected on the basis of relevance to subject matter.

**Results:**

In this review, we first introduce the influenza virus, its nomenclature, and the concepts of antigenic drift and shift. Second, we discuss the status of currently licensed influenza virus vaccines. We briefly focus on influenza vaccine responses beyond hemagglutination inhibition that may correlate with protection against influenza viruses of different subtypes. Third, we explain how studying host responses to influenza infection and vaccination with advanced serologic methods, B-cell receptor sequencing, and transcriptomic profiling can guide the development of improved influenza virus vaccines. Fourth, we provide 2 suggestions on how current influenza vaccines can be optimized by redirecting immune responses toward conserved viral antigens and the use of adjuvants.

**Conclusion:**

Influenza vaccine design can benefit from novel insights obtained from the study of host responses to influenza virus infection and vaccination. Integration of the large amount of available clinical and preclinical data requires systems approaches that can elucidate novel correlates of protection and will guide further development of influenza vaccine.

**Key Messages**

• Influenza is a major public health concern.
• Influenza is a vaccine-preventable disease.
• Currently, licensed influenza vaccines need yearly reformulation and come with variable vaccine efficacy.
• Host immune responses to influenza virus infection or influenza vaccination can guide influenza vaccine development.

## Introduction

Influenza is a respiratory illness caused by influenza viruses. It is a major public health concern with a huge economic impact worldwide.^1^, ^2^, ^3^ Vaccines against influenza virus are the best method of protection from influenza. Very young people, elderly population, pregnant women, and immunocompromised individuals are at enhanced risk for severe complications during infection. Therefore, these individuals form special target groups for influenza vaccination. Because of the increase in life expectancy, the elderly population is increasing in countries with an aging population. Despite being a vaccine-preventable disease, the protective effect of seasonal influenza vaccines is in general short-lived. Mutations in the influenza main surface antigenic determinants allow the virus to escape vaccine-induced neutralizing antibodies. Moreover, a decrease in the vaccine-induced antibody levels over time has been reported.^4^ There is a large window for improvement of influenza vaccines to provide better and longer protection against antigenically diverse influenza viruses. In this review, we briefly discuss the currently licensed influenza vaccines and how lessons learned from the study of interactions between influenza virus and its host can guide the development of current and future influenza vaccines.

## Influenza Virus and Currently Licensed Vaccines

### Influenza Viruses, Nomenclature, and Antigenic Drift and Shift

**Figure 1:**
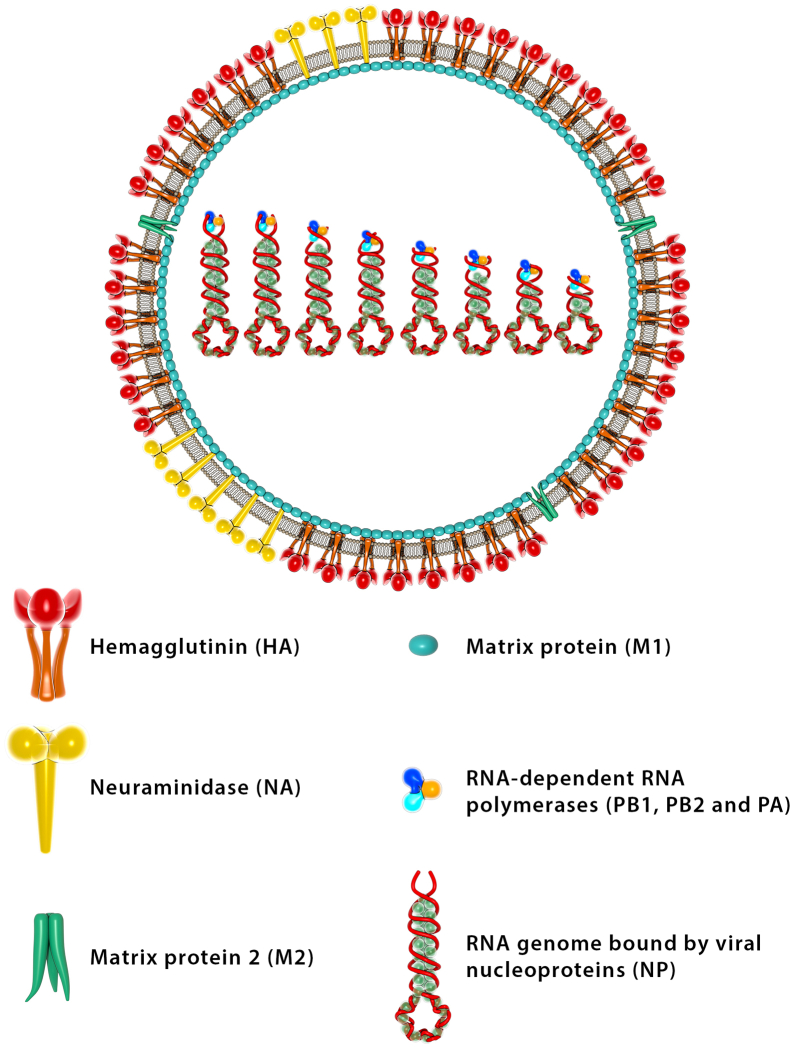
Schematic representation of influenza A virion. Influenza A viruses are enveloped negative-sense RNA viruses. The 2 major glycoproteins on the surface are hemagglutinin and neuraminidase. Also embedded on the surface of the virion envelope is the matrix protein 2. Underneath the envelope lies the matrix protein 1. Inside the virion are the 8 segmented negative-stranded RNA genomes that are bound by viral nucleoprotein to form ribonucleoprotein complexes. The 3 polymerase subunits (PB1, PB2, and PA) are assembled at the genomic RNA termini. Each segment encodes for 1 or 2 viral proteins. PA, polymerase acidic protein; PB1, polymerase basic protein 1; PB2, polymerase basic protein 2.

Influenza A and B viruses are the leading causes of epidemics in the human population. All influenza viruses contain a negative-sense, single-stranded segmented RNA genome. Each segment encodes for 1 or 2 viral proteins. The 2 major glycoproteins on the surface of the virus are hemagglutinin (HA) and neuraminidase (NA) (Fig 1). On the basis of these surface proteins, influenza A viruses can be further classified into different subtypes. Through serologic characterization, there are 18 antigenically different HAs and 11 antigenically diverse NAs. Different combinations of HA and NA subtypes can be found circulating in animals, such as avian and swine species. These different strains are classified in the format HxNy, where x and y are numbers that refer to the specific HA and NA subtypes. Currently in humans, only H1N1 and H3N2 are cocirculating. Unlike influenza A, influenza B viruses are not divided into subtypes. They belong to 2 antigenic lineages, which are historically called the B/Yamagata and B/Victoria lineages. Both lineages also cocirculate in the human population.

The World Health Organization recommends a specific nomenclature system for influenza viruses.^5^ Names are given in the order of type of influenza virus, host species (if not human), geographic location from which the virus was isolated, and finally the strain number and year of isolation. For A viruses, the subtypes are indicated after the viral isolate name in parentheses. For example, A/New Caledonia/20/1999 (H1N1) refers to an influenza A virus isolated from a person in New Caledonia in the year 1999 and assigned the strain number 20. This isolate also contains an H1 HA and N1 NA.

**Figure 2:**
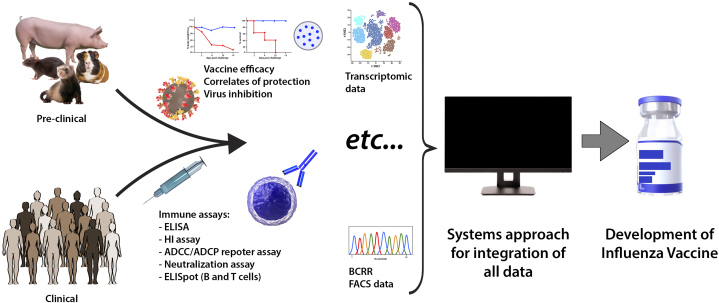
Study of host-virus and host-vaccine responses can drive influenza virus vaccine development. Clinical and preclinical studies that focus on host-virus and host-vaccine immune responses produce vast amounts of data. The availability of systems approaches allows the definition of correlates and surrogates of protection with input of multiple parameters, which provides useful information for influenza vaccine development. ADCC, antibody-dependent cellular cytotoxicity; ADCP, antibody-dependent cellular phagocytosis; BCRR, B cell receptor repertoire; ELISA, enzyme-linked immunosorbent assay; ELISpot, enzyme-linked immune absorbent spot; FACS, flow cytometry; HI, hemagglutination inhibition.

The influenza HA is the major surface antigenic determinant of influenza viruses. On exposure to the virus, the host antibody response targets a limited number of specific immunodominant sites on the HA.^6^ However, because of the virus’ ability to accumulate mutations at antigenic sites during replication (antigenic drift), influenza virus can escape preexisting immunity acquired by vaccination or infection during previous influenza seasons. Thus, annual influenza outbreaks still occur. The segmented nature of the genome (Fig 2) also allows the exchange of whole gene segments when influenza viruses of different subtypes simultaneously infect a susceptible host cell (antigenic shift). Therefore, this phenomenon can result in the emergence of a new influenza virus with surface antigens for which no preexisting immunity is present in the human population. These novel reassortant influenza viruses may originate from the animal reservoir as zoonotic infections. For example, pigs can act as a mixing vessel because they can be infected by avian and human influenza viruses.^7^ When such reassortant viruses obtain the ability to efficiently transmit from one human to another, the next pandemic can start.

### Currently Licensed Influenza Vaccines

Vaccines are the most effective way to provide protection and control against the seasonal influenza virus. More than 150 million doses of seasonal influenza vaccines are produced annually in the United States alone.^8^ There are 3 classes of vaccines that are being used: inactivated virus, live attenuated virus, and recombinant HA influenza vaccines. Influenza vaccines are trivalent (containing an H1N1 strain, an H3N2 strain, and 1 B virus lineage) or quadrivalent (containing both H1N1 and H3N2 strains and both B lineages). Each year, the World Health Organization predicts the next circulating viruses and provides seed strains for vaccine production. In the past, these seed strains were derived from coinfection cultures in which the new seasonal influenza virus strain was allowed to exchange gene segments encoding surface HA and NA with the gene segments encoding the internal proteins of a vaccine production virus. The acceptance of recombinant DNA techniques for the production of new reassortant vaccine strains made coinfection cultures unnecessary and has allowed for more efficient seed strain production.^9^ This is important because production speed is an important factor when fighting a seasonal virus.

In the case of inactivated influenza virus vaccines, the virus is typically split into individual proteins with detergent after inactivation, for example, by formaldehyde treatment. Split vaccines can be further enriched for the HA and NA proteins to provide subunit vaccines. Live attenuated vaccines are administered intranasally and rely on cold-adapted influenza viruses that do not replicate well in the lower respiratory tract and therefore do not cause disease. This attenuated phenotype is due to mutations in viral genes other than HA and NA. Similar to the inactivated vaccines, live attenuated vaccines are reformulated yearly to match the HA and NA of circulating strains. They are less used than inactivated vaccines and only effective in children because preexisting influenza virus immunity in adults limits the replication and immunogenicity of the live attenuated virus. Recombinant HA that contains influenza virus vaccines produced in insect cell cultures are also approved, but their use is still limited.

Currently, licensed seasonal influenza vaccines are formulated to induce neutralizing antibodies that target virus strain–specific HA on the surface. However, predicting the next circulating strain is difficult, and sometimes the HA of the circulating virus undergoes antigenic drift and shift and antigenically mismatches the HA contained in vaccine strains. In such cases, vaccine-induced antibodies will have reduced effectiveness in targeting the circulating virus, and efficacy of the vaccine can be low. Another cause of antigenic mismatch between vaccine and circulating virus originates from the vaccine production process. Many of the influenza vaccines currently in use are produced on embryonated chicken eggs. However, mutations in the HA as a result of egg adaptation have resulted in vaccine failure in the recent past.^10^

Another weakness of current vaccines is that in the very young and elderly populations, increasing protective immune responses by a single vaccination is often unsuccessful. Young children typically have no preexisting immunity against influenza virus after maternal antibodies wane. Therefore, 2 consecutive influenza vaccinations should be given in a pediatric setting to induce protective antibody levels.In contrast, the older population has preexisting immunity against influenza; however, both innate and adaptive immune responses in older adults are affected by immunosenescence. As a result, immunosenescence is thought to be responsible for high morbidity and mortality in elderly adults after influenza virus infection. To overcome this inefficient induction of influenza-specific antibodies in the elderly population, high-dose vaccines with twice the HA content of conventional vaccines or MF59-adjuvanted vaccines are available for this group.^11^, ^12^, ^13^ As we discuss in more detail below, studies on the interactions between the host and vaccine or influenza virus can help steer vaccine development to boost immune responses in specific populations.

The production and licensure process of current influenza vaccines is too slow for dealing with a seasonal or pandemic influenza virus outbreak. It typically requires 6 months for vaccine manufacturers and authorities to have an influenza vaccine mass produced and ready for distribution. This means that during an influenza pandemic, similar to the one caused by swine-origin H1N1 virus in 2009,^14^ the human population is left unprotected during the critical early months of the outbreak. The duration of 6 months is also long for a seasonal virus because vaccine updates are required annually. There are multiple time-limiting factors in this process, such as efficiency of current influenza vaccine production, formulation, and distribution methods. In addition, regulations and availability of reagents related to vaccine evaluation can delay licensure and release by the authorities.^15^ The time pressure would be much less for vaccine manufacturers and authorities if influenza vaccines can provide longer protection and do not require annual updates.

Given the economic and public health burden influenza viruses cause and the current status of licensed influenza vaccines for humans, it is clear that there is a need for better influenza vaccines. The National Institute for Allergy and Infectious Diseases (NIAID) has made it a priority to develop a universal influenza vaccine with the ability to not only provide protection during epidemics caused by seasonal influenza strains but also prevent major diseases during pandemics. In addition, NIAID published a strategic plan that was written to coordinate the scientific community’s efforts in universal influenza vaccine development.^16^ A part of NIAID’s plan focuses on characterization of the host immune response to influenza vaccination and infection to come up with new immune correlates of protection and support rational influenza vaccine design. We give some examples of how recent developments in the immunology field can contribute to the development of new influenza vaccine approaches.

## Learning From Host-Pathogen and Host-Vaccine Interactions to Steer Influenza Vaccine Development

### What Is the Preferred Type of Response to Influenza Vaccination?

For vaccines to work, induction of a protective response (quality) that is long-lasting (memory) is crucial. As discussed previously, the ever-changing antigenic nature of influenza viruses makes this challenging. To deal with viral escape from neutralizing antibodies, a promising approach is to focus on more conserved epitopes of influenza viruses. By doing so, the protective scope of vaccines can be extended to different types and subtypes of influenza viruses. This extension is needed for pandemic preparedness and to prolong efficacy of seasonal vaccines to cope with the antigenic drift in circulating viruses. Both the humoral and cellular branches of the adaptive immune system can target conserved influenza antigens and cross-react with antigens of different influenza virus subtypes.

Cross-reactive cytotoxic T cells can recognize epitopes contained in the more conserved internal viral antigens, such as matrix 1 protein and nucleoprotein, because T-cell epitopes are presented in the context of molecules of the major histocompatibility complex. Influenza virus–specific cytotoxic T cells kill infected epithelial cells after recognizing the major histocompatibility complex–epitope complex with its T-cell receptor. Because cytotoxic T cells target infected cells and not the virus directly, T-cell–mediated immunity typically is infection-permissive, allowing some degree of virus replication. T-cell recognition of conserved viral antigens presented by antigen-presenting cells can also contribute to a more qualitative antibody response, for example, by promoting IgG class switching in germinal centers. Clinical challenge studies have found that both CD4^+^ and CD8^+^ T cells can correlate with protection against influenza.^17^, ^18^, ^19^ However, current licensed vaccines are poor inducers of efficient T-cell responses.^20^

Induction of antibodies that target conserved influenza antigens is high on the list of vaccine responses with the potential to provide broad protection. Virus-neutralizing antibodies usually target the virus directly, for example, on the globular heads of HA or NA. However, antibodies that bind viral antigen on the surface of infected cells can also contain viral infection by interfering with the viral release and enhancing clearance of infected host cells. Therefore, induction of non-neutralizing cross-reactive antibodies by influenza vaccination can contribute to vaccine efficacy and protection.

Finally, induction of a favorable innate immune environment plays a crucial role for a qualitative, long-lasting vaccine response. Therefore, vaccination protocols should also consider induction of favorable innate immune responses to optimize vaccine responses. In summary, because of the mutagenic nature of influenza virus, we need vaccines that can induce broad and long-lasting immune responses. Therefore, many studies are ongoing to find methods to achieve this goal. Taking advantage of conserved epitopes of influenza viruses has been one of the many methods to induce cellular and humoral immune responses that cross-react with different influenza types and subtypes. Other than the adaptive immune mechanism, the innate immune responses can also provide an environment that allows enhancing the longevity and boosting of immune responses toward the influenza virus.

### The Challenge of Finding Good Immune Correlates of Protection

The current gold standard correlate of protection is the hemagglutination inhibition (HI) titers in sera.^21^ The HI assay serves as a proxy for virus neutralization by antibodies that prevent the virus to bind to its host cell receptor. This in vitro assay has been widely adopted as an immune correlate of protection, but it has been contested in the influenza vaccine field. A vaccine response resulting in an HI titer of 40 or a 4-fold increase of the initial HI titer is considered to be 50% protective. However, multiple components of the host immune response to infection and vaccination can contribute to protection against influenza virus infection, and the HI assay does not measure all these immune responses. The same concern can be raised for other antibody-based in vitro assays based on virus neutralization, such as microneutralization assays. Multiple immune mechanisms that do not result in virus neutralization can contribute to heterosubtypic immunity against influenza viruses of different subtypes. As discussed previously, cross-reactive T cells that target conserved influenza virus antigens provide protection without complete virus neutralization. Ways by which the humoral branch of the vaccine response can also contribute to protection beyond virus neutralization is through Fc receptor engagement^22^ followed by phagocytosis (antibody-dependent cellular phagocytosis) or killing of the infected cell (antibody-dependent cellular cytotoxicity), and complement fixation. Therefore, it is important that other immune correlates of protection are considered.

Some measurable immune parameters correlate with protection without revealing the protective mechanism. For example, it is possible that enzyme-linked immunosorbent assay (ELISA) antibodies against influenza antigens correlate with protection. However, ELISA is only informative on the antigen that is targeted by the antibody and not how it protects. Therefore, antibodies analyzed through ELISAs are sometimes called surrogates of protection. Different assays that consider immune markers beyond neutralization have been proposed to find new correlates that predict efficacy for influenza vaccines.^23^

To assess the protective ability of influenza virus vaccines, an assay is needed to correlate vaccine responses with protection against the virus. Although HI assays have been established as the gold standard correlate of protection for currently licensed influenza vaccines, HI assays do not consider other immune mechanisms beyond virus neutralization. This consideration is important because a variety of immune mechanisms, other than neutralizing the virus directly, can play a role in protection. Thus, to guide future influenza vaccine development, a good understanding of immune correlates and surrogates of protection is necessary.

### Examples of Recent Technologies to Study Host-Virus and Host-Vaccine Responses of Importance for Future Influenza Vaccine Development

#### Advanced Serologic Analysis to Detect Cross-reactive Antibodies Targeting Conserved Epitopes

Influenza virus infection can result in antibody responses that cross-react with HAs from different subtypes.^24^^,^^25^ This cross-reaction suggests that natural infection can induce antibody responses that target conserved epitopes in influenza virus antigens. Nevertheless, repeated infections with influenza viruses are common, and therefore, these cross-reactive antibodies may not provide complete virus neutralization and absolute protection against reinfection. However, in combination with cross-reactive T cells, non-neutralizing antibodies can form a layer of preexisting immunity, preventing lethality while still allowing some degree of morbidity after infection.

In a longitudinal family cohort study, antibodies that target the conserved HA stalk have been found to correlate with protection, in addition to NA antibodies.^26^ Recently, cross-reactive antibodies that target conserved sites on the influenza NA protein have also been found in both mice and humans,^27^, ^28^, ^29^ illustrating the potential of NA as a vaccine antigen. Although current seasonal vaccines contain some amount of NA, the quality and quantity in the vaccine are not standardized like HA. In addition, HA is immunodominant to NA.^30^ In combination, this immunodominance results in poor anti-NA antibodies after vaccination. Therefore, many groups are working to increase the immunogenicity of NA through development of new vaccine formulations and strategies.^31^, ^32^, ^33^ Similarly, antibodies that target the conserved ectodomain of the influenza matrix 2 protein (M2e) were protective in preclinical and clinical settings.^34^ Because of its abundance on the surface of the virus, HA has been the most attractive vaccine target. Moreover, HA can be divided into the head domain and the stalk domain. Although the HA head domain is variable, there are still highly conserved regions in the head domain.^35^, ^36^, ^37^ As discussed briefly, the stalk domain of HA is also conserved. Thus, many strategies have been developed to increase immunogenicity toward these conserved regions of HA.^38^ As a result, studies have found that antibodies against these conserved regions in the HA can provide broad protection against different influenza viruses. Vaccination strategies are currently ongoing to make these conserved influenza antigens more immunogenic by redirecting antibody responses from the immunodominant but variable epitopes in the HA head toward more conserved epitopes in HA and other viral antigens.^34^^,^^39^^,^^40^

Recent technological advances allow the investigation of cross-reactive sera with unforeseen resolution. With recombinant technologies, influenza virus proteins can be produced with high purity. Other than using these recombinant proteins as a vaccine antigen, they can be used to run various assays to further identify and characterize the functions of antibodies that bind to specific proteins of influenza virus. For example, the availability of recombinant influenza proteins allows researchers to sort cross-reactive plasma cells through flow cytometry. The B-cell receptor encoding genes can then be sequenced to produce recombinant cross-reactive antibodies. These antibodies can be used to further investigate the individual contribution of a single antibody to vaccine efficacy and define the phenotype of the B cell or plasma cell from which it originated.^41^^,^^42^ Such an investigation also allows the identification of rare cross-reactive B-cell clones for further in-depth characterization.^29^ Technological progress in the fields of monoclonal antibody production and protein crystallography has allowed relatively fast analysis of how and where infection-induced (cross-reactive) serum antibodies bind viral antigen.^37^^,^^43^ In addition to being used as tools for vaccine design, these antibodies can be used as therapeutics for high-risk groups and medical personnel during pandemics. Several monoclonal antibodies are in clinical trials and have had promising results.^44^ The abundance of data obtained from such studies can be further combined with other antibody features, such as binding affinities and specific Fc functions (eg, antibody-dependent cellular cytotoxicity and antibody-dependent cellular phagocytosis), using a systems approach called *systems serology*.^45^ The in-depth analysis of humoral responses with systems serology can guide future influenza vaccine development by suggesting which type of humoral response is to be favored by defining new correlates of protection based on multiple mechanisms of protection.

Targeting conserved epitopes on the influenza virus is one strategy to induce cross-reactive antibodies. Using information collected through advanced technology, specific monoclonal antibodies and new target epitopes can be discovered to develop better vaccines and therapeutics. In addition, with the help of systems serology, mechanisms of protection can be further analyzed to find better correlates of protection provided by humoral immune responses.

#### Antigen-Specific B-Cell Receptor Repertoire Sequencing to Develop Vaccines That Can Skew Toward Preferred Epitopes

Antigen-specific recognition of viral and other antigens by lymphocytes is a cornerstone of adaptive immunity. Maintenance of a diverse B-cell receptor repertoire allows recognition and acquisition of pathogen-specific immunity that can protect during the initial infection or reinfection later in life. Somatic V, D, and J segment rearrangement results in diverse B-cell receptor repertoires.^46^ On antigen recognition, clonal selection of lymphocytes with a unique antigen-engaged receptor can occur. By clonal expansion of epitope-specific B cells, the receptor repertoire is shaped in an antigen-specific manner. Further diversification of the B-cell receptor repertoires after VDJ rearrangement occurs by somatic hypermutation of receptor-encoding genes during clonal expansion of antigen-specific lymphocytes.^47^

High-throughput sequencing of B-cell receptor genes has provided information on the clonality and diversity of immune repertoires. Single-cell RNA sequencing especially allows the determination of the combination of heavy and light antibody chains for individual B cells. The importance of studying the lymphocyte receptor repertoire for vaccine development has become clearer in the past decade. Existing sequencing efforts reveal that although in theory endless complexity is possible, receptor repertoire diversity and size are defined by certain constraints. For example, Jackson et al^48^ observed the presence of influenza-specific signatures of convergent antibody heavy chain rearrangements on pandemic influenza H1N1 infection or vaccination in different individuals. A strong correlation between B-cell clonal expansion in blood and the serologic response to influenza vaccination was also observed in the same study. The ability to link B-cell receptor repertoires to antigen specificities allows the probing for new influenza vaccine targets with the ability to induce clonal B-cell expansion, such as the conserved influenza epitopes that are interesting for universal influenza vaccine development.^49^ Investigation of the influenza-specific B-cell repertoire can also help in the better understanding of the potential negative effect of preexisting immunity to influenza virus on influenza vaccine efficacy when recall of dominant B-cell clones that target the old influenza antigen interferes with the induction of de novo B-cell responses toward the updated influenza vaccine.^50^ Guided by the presence of antigen-specific convergent B-cell receptor repertoires and detection of subdominant B-cell clones with specificity for conserved epitopes, we will be able to choose or modify influenza vaccines such that vaccine responses are optimally skewed toward the preferred epitopes, for example, the stalk of HA.^49^^,^^51^, ^52^, ^53^

To achieve the goal of broad protection against influenza virus, targeting conserved epitopes is a major objective. Therefore, with the help of single-cell sequencing technology, vaccination strategies can be developed to induce specific humoral responses that can provide broad protection.

#### Importance of Postvaccination Transcriptomic Profiling in the Innate Immune Response for Vaccine Responses

The induction of protective, long-lasting adaptive immune responses is the aim of vaccination. The quality and magnitude of adaptive immune responses are controlled by the innate immune response that precedes the adaptive immune answer.^54^ After sensing pathogen-associated molecular patterns, innate immune cells, such as dendritic cells, are activated. This activation results in cytokine production, such as interferon type 1 (IFN-1), and the establishment of a favorable immune environment to initiate adaptive immune responses. Transcriptomic profiling of postinfluenza vaccination immune responses has revealed gene signatures and modules that correlate with and are even predictive for the vaccine antibody response.^55^^,^^56^ For example, Nakaya et al^55^ found that trivalent inactivated influenza vaccine vaccinees had a higher number of differentially expressed genes related to antibody-secreting cells. These gene signatures related to antibody-secreting cells were up-regulated and predictive of HI titers at 28 days after vaccination. During the earlier phases of the vaccine response, vaccine-induced gene signatures were related to the early innate immune response driven by IFN-1–associated genes^57^ and provide an explanation for the differences in vaccine efficacy for different types of influenza vaccines or in different age groups.^58^^,^^59^ These results illustrate the power of transcriptomic profiling for evaluation of influenza vaccine responses and unravel the mechanisms of action influenza vaccines can rely on to induce a protective immune response. One of these mechanisms is the induction of an IFN-1–driven innate immune response immediately after vaccination.

Although adaptive immune response has been the main focus of vaccine development, these transcriptomic studies have proved the importance of innate immune responses after vaccination and infection. Thus, with the use of these studies, strategies can be developed to trigger specific innate pathways that can lead to stronger adaptive immune responses that are protective against infection.

### Optimization of Current Licensed Influenza Vaccines

#### Redirecting Immune Responses From Variable Immunodominant Regions to More Conserved Epitopes

Currently licensed influenza vaccines are normalized for the HA content of the different influenza virus strains they contain. As discussed previously, the viral NA is a promising vaccine antigen with the potential to provide protection against drifted influenza strains. To further exploit the NA as a vaccine antigen, vaccines should be standardized for NA content as well. The preference for ELISA-based assays for standardization is because assays based on NA activity will not discriminate among NAs from different virus strains in the vaccine.^32^ Moreover, NA activity can be lost during the production process of inactivated influenza viruses or because of protein instability during storage. Another option to enhance content of more conserved antigens that has been explored in preclinical models is to supplement conventional influenza vaccines with soluble forms of NA or recombinant forms of the M2e.^60^, ^61^, ^62^

Many universal influenza virus vaccine approaches are being developed that target conserved epitopes. One method that is currently being tested in clinical trials is to redirect antibody responses toward the immunosubdominant stalk domain of HA, which is more conserved than the head domain of HA. Although this approach is not based on a currently licensed influenza vaccine, we refer to it because it uses the same production platform (split and live attenuated influenza vaccine).^63^

#### Adjuvants

Adjuvants enhance the immunogenicity of influenza vaccines, thereby enhancing vaccine efficacy and effectiveness. An important advantage of adjuvant-mediated enhanced immunogenicity is that it allows vaccine dose sparing, which is important during times of vaccine shortage, for example, during a pandemic. Currently, squalene-based oil-in-water adjuvants, such as MF59 and AS03, are used for immunopotentiating influenza vaccines. Recent insights from clinical data obtained for oil-in-water adjuvanted influenza vaccines suggest that adjuvants promote and skew B-cell responses in multiple ways: increasing activation of naive B cells, increasing the adaptability of memory B cells during antigenic recall, and promoting vaccine-mediated T-cell induction, which in turn can support class switching of B cells.^64^^,^^65^ Currently used adjuvants were developed empirically, and the mechanisms that these adjuvants exploit to enhance and broaden vaccine responses are not fully understood. Adjuvants typically trigger innate immune responses at the site of administration, which is important for the subsequent induction of the adaptive immune response, as we have discussed more in depth previously.^66^ Therefore, vaccinology in general and influenza vaccination in specific will benefit from advances in the field of innate immune signaling.^67^, ^68^, ^69^ The discovery of new agonists of innate immune sensors will feed the pipeline of novel adjuvant candidates that allow the fine-tuning of the innate immune environment, resulting in optimal vaccine responses. Therefore, adjuvants are attractive tools to enhance effectiveness of currently available influenza vaccines.

In addition to developing a better and novel influenza virus vaccine, studies in optimizing currently licensed influenza vaccines are ongoing. Inducing antibodies against nucleoprotein, M2e, and NA can increase the effectiveness of current seasonal vaccines. Standardizing the amount of other viral proteins, other than HAs, in vaccine manufacturing can help address this issue. In addition, the use of adjuvants with current vaccines can help boost immune responses. Although there are a variety of adjuvants in development or approved for use in humans, there remains a need for testing them in combination with influenza vaccines to enhance antibody responses and help generate stronger cellular immune responses. Further understanding the virus-host interaction can help discover new targets on the host that can lead to novel adjuvant candidates.

## Conclusion

Although we are on the road toward a true universal influenza vaccine to be available in the pharmacy, currently licensed vaccines are the best option to protect against influenza virus infection. Through the addition of adjuvants or new vaccine production platforms, current licensed seasonal vaccine approaches are being upgraded slowly. Although not discussed in detail in this review, development of new vaccine production platforms has helped with increasing immune responses that target influenza virus. Epitope-based vaccines try to focus the immune response toward epitopes that are crucial for the virus but might not be immunogenic by nature. The HA stalk domain discussed elsewhere in this review is such an epitope, and targeting host immune responses against conserved epitopes with epitope-based vaccines is often used to broaden the specificity of the vaccine response.^39^ We briefly want to mention messenger RNA– and DNA-based vaccines, 2 vaccine platforms that have generated great interest because they can be produced in mass amounts in shorter time compared with the conventional approaches.^70^

Influenza vaccine development is hampered to some extent by the fact that only a limited number of correlates or surrogates of protection are used to predict vaccine efficacy.^71^ The preclinical and clinical data gathered from animal models and human cohort studies performed with currently licensed influenza vaccines or after influenza infection are crucial for gaining a better understanding of immune correlates of protection. Anti-influenza immunity is a complex system shaped by interactions between innate and adaptive immune responses at different levels and influenced by the previous immune history of the individual, including first and subsequent exposures to influenza virus infections and vaccinations. It is likely that a combination of immune correlates or surrogates of protection for influenza virus will have more predictive value for vaccine efficacy, especially if the influenza vaccine relies on correlates of protection different from the currently accepted ones. The size and complexity of data sets that become available require highly advanced data management systems, storage capacity, systems biology approaches, and machine learning for analysis and prediction, which would allow for a better understanding of the influenza vaccine response. With new technologies, development of a more targeted influenza vaccine can be established and will be guided by novel immune correlates of protection that we are starting to understand better. The challenge is now to put all available data at work to optimize current and future influenza vaccine approaches that can lead to the development of an influenza vaccine that can protect the human population from the burden influenza viruses still cause.
